# Clinical biomarkers for thyroid immune-related adverse events in patients with stage III and IV gastrointestinal tumors

**DOI:** 10.3389/fimmu.2024.1381061

**Published:** 2024-05-07

**Authors:** Na Xing, Jing Liu, Lin Hou, Yue Zhao, Hongfang Ma, Fujun Wang, Zhanjun Guo

**Affiliations:** ^1^ Department of Rheumatology and Immunology, The Fourth Hospital of Hebei Medical University, Shijiazhuang, China; ^2^ Department of Endocrinology, The Fourth Hospital of Hebei Medical University, Shijiazhuang, China; ^3^ Department of Cardiology, The Fourth Hospital of Hebei Medical University, Shijiazhuang, China; ^4^ Department of Gastroenterology, The Fourth Hospital of Hebei Medical University, Shijiazhuang, China

**Keywords:** thyroid, immune related adverse events, programmed cell death protein 1 inhibitors, adenosine deaminase, clinical biomarkers

## Abstract

**Background:**

Thyroid immune-related adverse events (irAEs) associated with immune checkpoint inhibitor (ICI) treatment appear to correlate with a better prognosis. We aimed to investigate clinical biomarkers associated with thyroid irAEs.

**Methods:**

We retrospectively analyzed data from 129 patients receiving programmed cell death protein 1 (PD-1) inhibitors for stage III and IV gastrointestinal tumors. Patients were divided into two groups: “thyroid irAEs” group and “no thyroid irAEs” group. We compared continuous variables using Mann–Whitney U and Kruskal–Wallis tests and categorical variables using Pearson’s chi–square test. Survival curves were generated using the Kaplan–Meier method, and associations between clinical features and thyroid irAEs were assessed using univariate and multivariate logistic regression models. Associations for thyroid irAEs and outcomes [progression-free survival (PFS), overall survival (OS)] of the patients were performed with a Cox proportional hazard model.

**Results:**

A total of 129 patients, including 66 gastric cancer, 30 esophageal squamous cell carcinoma, and 33 hepatocellular carcinoma (HCC), were involved in this analysis with 47 cases of thyroid irAEs occurrence. The Cox proportional hazard model analysis confirmed the extended PFS [hazard rate (HR) = 0.447, 95% confidence interval (CI): 0.215 to 0.931, *p* = 0.031] and OS (HR = 0.424, 95% CI: 0.201 to 0.893, *p* = 0.024) for thyroid irAEs group when compared with those of the no thyroid irAEs group. Association between thyroid irAEs and clinical characteristics at baseline was analyzed subsequently by univariate analysis. Higher body mass index (*p* = 0.005), increased eosinophil count (*p* = 0.014), increased lactate dehydrogenase (*p* = 0.008), higher baseline thyroid stimulating hormone (TSH) (*p* = 0.001), HCC (*p* = 0.001) and increased adenosine deaminase (ADA) (*p* = 0.001) were linked with thyroid irAEs occurrence. The multivariable logistic regression model indicated that ADA [odds rate (OR) = 4.756, 95% CI: 1.147 to 19.729, *p* = 0.032] was independently associated with thyroid irAEs occurrence.

**Conclusions:**

Increased baseline level of ADA was associated with thyroid irAEs occurrence in patients with advanced gastrointestinal tumors who received ICI treatment. In the case of abnormal ADA, attention should be paid to the risk of thyroid irAEs.

## 1 Introduction

The global incidence and mortality rates of gastrointestinal tumors in China have reached approximately 50% (1), indicating the necessity for increased attention toward their prevention, diagnosis, and treatment. Since 2011, immune checkpoint inhibitors (ICIs), which promote the anti-tumor immune response of activated T cells by blocking the inhibitory signal, including cytotoxic T-lymphocyte associated protein 4 inhibitors, programmed cell death protein 1 (PD-1) inhibitors, and programmed cell death ligand 1 inhibitors, have been used as a treatment method for unresectable or metastatic melanoma, lung cancer, breast cancer, and gastrointestinal tumors with monotherapy or combined therapy (2). Immune-related adverse events (irAEs) occur owing to the overactivation of the immune system following ICI treatment. This overactivation triggers enhanced T cell activity towards antigens found in both tumor and normal tissues, inducing an inflammatory response and alterations in the autoimmune environment, thereby initiating the incidence of irAEs (3, 4). IrAEs appear to be associated with a favorable prognosis for ICI treatment, however, severe adverse reactions may necessitate discontinuation of medication and can even be life-threatening (2).

IrAEs can affect various organs and systems, causing dysfunction; thyroid dysfunction is a common endocrine irAE with a widely range of incidence rates in different reports (5–7). The mean incidence rate in phase III clinical trials is 10.8%, especially in patients using anti-PD1 drugs (8). Thyroid dysfunction includes primary hyperthyroidism, primary hypothyroidism, central hypothyroidism, and biphasic thyroiditis that transitions from transient hyperthyroidism to hypothyroidism with severe grade in rare cases (6). Thyroid dysfunction as an irAE has been associated with prolonged survival in various cancers, including caseds of non-small cell lung carcinoma (NSCLC), malignant melanoma, renal cell carcinoma, head and neck cancer, and gastric cancer (4–6). However, thyroid dysfunction can lead to metabolic abnormalities affecting the overall treatment effectiveness.

Few studies have focused on clinical biomarkers for the occurrence and timely monitoring of thyroid dysfunction as an irAE (5). High baseline levels of thyroid stimulating hormone (TSH) and positive thyroid related antibodies were associated with the occurrence of thyroid irAEs, but others biomarkers didn’t reach consensus. This study is aimed to investigate clinical biomarkers for earlier diagnosis and follow-up of thyroid irAEs. Adenosine deaminase (ADA) is associated with the immune status in patients with solid tumors, with alterations observed during thyroid dysfunction occurred. ADA is a key enzyme regulating purine metabolism by degrading adenosine, thereby reducing its inhibitory effects on the immune system. Currently, no research has focused on the role of ADA in the occurrence of irAEs. In this study, we aimed to identify clinical biomarkers for the occurrence of thyroid irAEs.

## 2 Materials and methods

### 2.1 Patients

Patients diagnosed with stage III and IV gastrointestinal tumors and undergoing treatment with anti-PD-1 antibody monotherapy or combined therapy (chemotherapy or targeted therapy) at The Fourth Hospital of Hebei Medical University from November 2018 and November 2021 were included in this study. The inclusion criteria were: (1) any cancer type treated with a minimum of two courses of ICIs; (2) availability of serum thyroid hormone evaluation at baseline; and (3) availability of at least two different serum thyroid hormone evaluations during ICI treatment. The exclusion criteria were: (1) a surgical history of thyroid disease; (2) a history of head and neck radiotherapy; (3) a history of pituitary disease; (4) abnormal baseline thyroid hormone, and (5) a history of treatment with amiodarone. Clinical information, including age, sex, body mass index (BMI), cancer type, Eastern Cooperative Oncology Group Performance Status (ECOG PS), TNM stage, treatment lines, treatment regimen, irAEs, baseline peripheral blood markers, baseline thyroid function tests, and posttreatment thyroid function tests, was obtained from electronic medical records. The National Cancer Institute Common Terminology Criteria for Adverse Events ver. 4.03 (https://ctep.cancer.gov/protocolDevelopment/electronic_applications/ctc.htm#ctc 40) were used for irAE assessment.

All procedures were approved by the Ethics Committee of the Fourth Hospital of Hebei Medical University. This retrospective study analyzed existing data, and the requirement for informed consent was waived.

### 2.2 Treatment and assessment

Patients received standard anti-PD-1 antibodies (sintilimab, camrelizumab, pembrolizumab, toripalimab, and tisleizumab) every 3 weeks as monotherapy or combined therapy (targeted therapy/chemotherapy) until disease progression, clinical worsening, unacceptable toxicity, or patient refusal. No cytotoxic T lymphocyte-associated antigen-4 inhibitor was used in these patients. Clinical and laboratory tests were performed during each cycle before dosing. Body computed tomography or magnetic resonance imaging scans were performed every 2–3 cycles. Progression-free survival (PFS) was defined as the time from the first administration of anti-PD-1 therapy to disease progression, death, or the study cutoff; overall survival (OS) was defined as the time from the commencement of anti-PD-1 therapy to death or the study cutoff values. PFS and OS were evaluated using the Response Evaluation Criteria in Solid Tumors version 1.1 as the study endpoints.

Patients were divided into two groups based on thyroid function outcomes: the “thyroid irAEs” group and the “no thyroid irAEs” group. Thyrotoxicosis was defined as a TSH level <0.34 mIU/L, with free thyroxine (FT4) or total thyroxine (TT4) levels within or above the reference range. Hypothyroidism was characterized by a TSH level above the upper reference range, with FT4 or TT4 levels within or below the lower reference interval. Biphasic thyroiditis indicated transient thyrotoxicosis transitioning into hypothyroidism during follow-up. Thyroid function was measured using established reference ranges: TSH, 0.34–5.6 mIU/L; FT4, 7.98–16.02 pmol/L; free triiodothyronine, 3.53–7.37 pmol/L; TT4, 69.71–163.95 nmol/L; total triiodothyronine, 0.92–2.38 nmol/L; anti-thyroid peroxidase antibodies (TPOAb), <9 IU/mL; and thyroglobulin antibody (TGAb), <4 IU/mL.

### 2.3 Statistical analysis

Continuous variables were compared using Mann–Whitney U and Kruskal–Wallis tests, while categorical variables were analyzed using Pearson’s chi–square test. Survival curves were drawn using the Kaplan–Meier method. The associations between clinical features and thyroid irAEs were assessed using univariate and multivariate logistic regression models. The association between the clinical features and survival was analyzed using the log-rank test. The associations between thyroid irAEs and outcomes (PFS, OS) of the patients were performed with a COX proportional hazard model. The receiver operating characteristic (ROC) curve analysis determined the best cutoff value. All results were considered statistically significant at *p <*0.05. Statistical analysis was performed using SPSS version 25.0 software (IBM Corp, Armonk, NY, USA), and figures were generated using GraphPad Prism version 9 (GraphPad Software, San Diego, CA, USA).

## 3 Results

### 3.1 Clinical characteristics analysis for thyroid irAEs and no-irAEs groups

In the cohort, 129 inpatients including 60 females and 69 males were involved in this study, with a median age of 63 years [interquartile range (IQR) 56–69 years], a median PFS of 146 days [95% confidence interval (CI): 181.44–241.21 days] and a median OS of 239 days (95% CI: 260.78–322.32 days). Gastric cancer was the most common type of tumor (n = 66, 51.2%), while 33 patients had hepatocellular carcinoma (HCC), and 30 patients had esophageal squamous cell carcinoma. Thyroid irAEs were observed in 47 patients (11 thyrotoxicosis, 27 hypothyroidism, 9 biphasic thyroiditis), with a median of 3 (IQR 2–5) cycles of treatment. Patients with HCC were more prone to thyroid irAEs compared with other cancer types. The baseline information is shown in **Table 1**.

**Table 1:** Characteristics of patients with and without thyroid immune-related adverse events (irAEs).

	Total N (%)	No thyroid irAEs No. (%)	Thyroid irAEs No. (%)	*p*
Total N	120 (100)	82 (63.6)	47 (36.4)	
Sex				
female	60 (46.5)	37 (45.1)	23 (48.9)	0.676
male	69 (53.5)	45 (54.9)	24 (51.1)	
Age(years)				
≤60	51 (39.5)	29 (35.4)	22 (46.8)	0.201
>60	78 (60.5)	53 (64.6)	25 (53.2)	
BMI (kg/m^2^)				
≤21.06	54 (41.9)	42 (51.2)	12 (25.5)	0.004
>21.06	75 (58.1)	40 (48.8)	35 (74.5)	
ECOG PS				
≤1	60 (46.5)	36 (43.9)	24 (51.1)	0.433
>1	69 (53.5)	46 (56.1)	23 (48.9)	
Treatment Line				
≤2	100 (77.5)	61 (74.4)	39 (83.0)	0.261
>2	29 (22.5)	21 (25.6)	8 (17.0)	
Anti-PD-1 antibodies				
Sintilimab	31(24.0)	20(24.4)	11(23.4)	0.867
Camrelizumab	51(39.5)	31(37.8)	20(42.6)	
Pembrolizumab	19(14.7)	11(13.4)	8(17.0)	
Toripalimab	6(4.7)	4(4.9)	2(4.3)	
Tisleizumab	22(17.1)	16(19.5)	6(12.8)	
Disease Status				
III	47 (36.4)	30 (36.6)	17 (36.2)	0.962
IV	82 (63.6)	52 (63.4)	30 (63.8)	
Tumor Types				
HCC	33 (25.6)	12 (14.6)	21 (44.7)	0.001
Esophageal squamous cell carcinoma	30 (23.3)	19 (23.2)	11 (23.4)	
Gastric cancer	66 (51.2)	51 (62.2)	15 (31.9)	
Concurrent therapy				
Chemotherapy drugs	55 (42.6)	40 (48.8)	15 (31.9)	0.062
Targeted drugs	74 (57.4)	42 (51.2)	32 (68.1)	
Other irAEs				
No	111 (86.0)	71 (86.6)	40 (85.1)	0.816
Yes	18 (14.0)	11 (13.4)	7 (14.9)	
TPOAb				
Negative	84 (80.0)	58 (85.3)	26 (70.33)	0.066
Positive	21 (20.0)	10 (14.7)	10 (29.7)	
TGAb				
Negative	84 (80.0)	56 (82.4)	28 (75.7)	0.414
Positive	21 (20.0)	12 (17.6)	9 (24.3)	
Clinical Baseline Value[Median (IQR)]				
NLR	3.47 (2.90)	3.24 (2.70)	3.57 (3.57)	0.391
PLR	181.15 (140.43)	181.36 (135.39)	177.91 (164.39)	0.483
Eosinophil count (× 10^9^/L)	0.08 (0.13)	0.08 (0.11)	0.09 (0.19)	0.145
ADA(U/L)	11.90 (7.55)	10.90 (5.10)	14.20 (11.28)	0.005
LDH(U/L)	182.00 (82.68)	175.00 (67.50)	192.30 (135.00)	0.004
Baseline TSH (mIU/L)	1.67 (1.37)	1.48 (1.05)	2.24 (1.44)	0.001

IQR, interquartile range; NLR, neutrophil-to-lymphocyte ratio; PLR, platelet-to-lymphocyte ratio; ADA, adenosine deaminase; LDH, lactate dehydrogenase.

### 3.2 Thyroid irAEs associated with disease control rate, PFS and OS

As shown in **Table 1**, BMI, tumor type, ADA level, lactate dehydrogenase (LDH) level, and baseline TSH level differed between the thyroid irAEs and no thyroid irAEs groups, demonstrating that the baseline status of these indicators affected the occurrence of thyroid irAEs. The distribution frequency of thyroid irAEs incidence was not different for these 5 antibodies (*p* = 0.867). In **Table 2**, the disease control rate (DCR) between the two groups was significantly different (*p*= 0.002), which was observed 85.1% (95%CI:74.9% to 95.3%) in the thyroid irAEs group and 61.0% (95%CI:50.4% to 71.5%) in the no thyroid irAEs group.

**Table 2:** Effect of thyroid immune-related adverse events (irAEs) on immune checkpoint inhibitor (ICI) efficacy.

	Total	No thyroid irAEs	Thyroid irAEs	*p*
PD	38	32	6	–
SD	59	34	25	–
PR	300	16	14	–
CR	2	0	2	–
ORR	24.8% (16.5–30.6%)	19.5% (10.9–28.1%)	34.0% (20.5–47.6%)	0.066
DCR	69.8% (61.6–76.9%)	61.0% (50.4–71.5%)	85.1% (74.9–95.3%)	0.004

SD, stable disease; PR, partial response; PD, progressive disease; CR, complete response; ORR, objective response rate; DCR, disease control rate.

**Figure 1:**
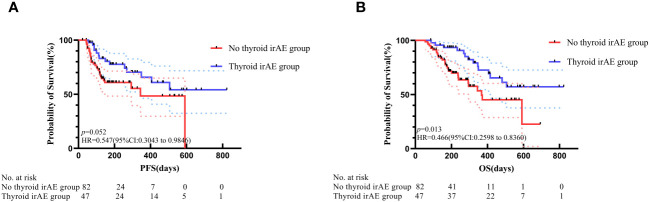
Association of thyroid irAEs with patient prognosis. **(A)** Kaplan–Meier curve showing PFS; **(B)** Kaplan-Meier curve illustrating OS.

The Kaplan–Meier curves of PFS and OS between the two groups are shown in **Figure 1**. The PFS analysis displayed a differentiated trend without statistical difference, while the median PFS in the thyroid irAEs group (559 [95% CI: 348–1142] days) was higher than that in the no-irAEs group (360 [95% CI: 247–441] days) (*p* = 0.052). The median OS in the no- irAEs group was 408 (95% CI: 235–499) days, which was lower than that (611 days [95% CI: 367–1123) days) in the thyroid irAEs group (*p* = 0.013). Univariate and multivariate analysis were performed for these clinical characteristics and outcomes (PFS and OS) in these patients. In **Table 3**, the multivariate analysis indicated the thyroid irAEs group [hazard rate (HR) = 0.447, 95%CI: 0.215 to 0.931, *p* = 0.031 for PFS; HR = 0.424, 95%CI: 0.201 to 0.893, *p* = 0.024 for OS] and treatment line (>2) (HR = 3.360, 95%CI: 1.592 to 7.092, *p* = 0.001 for PFS; HR = 2.137, 95%CI: 1.037 to 4.405, *p* = 0.040 for OS) were linked with outcome of these patients with gastrointestinal cancer. Higher level of neutrophil: lymphocyte ratio (NLR) was associated with a shorter OS at statistical levels (HR = 2.265, 95%CI: 1.138 to 4.508, *p* = 0.020). These data indicate that thyroid irAEs were associated with a better prognosis for these cancer patients after ICI treatment.

**Table 3:** Multivariate analysis of PFS and OS with COX regression models.

	PFS			OS		
	HR	95%CI	*p*	HR	95%CI	*p*
Age (years)						
≤60 >60	Reference 1.080	0.552-2.114	0.822	Reference 1.441	0.746-2.783	0.277
BMI (kg/m2)						
≤21.06	Reference		0.191	Reference		0.158
>21.06	0.645	0.334-1.244		0.626	0.326-1.201	
ECOG PS						
≤1 >1	Reference 1.577	0.778-3.189	0.207	Reference 1.587	0.785-3.206	0.198
Treatment Line						
≤2	Reference		0.001	Reference		0.040
>2	3.360	1.592-7.092		2.137	1.037-4.405	
Tumor Types						
HCC	Reference			Reference		
Esophageal squamous cell carcinoma	0.431	0.143-1.298	0.135	0.574	0.200-1.643	0.301
Gastric cancer	0.515	0.218-1.221	0.132	0.744	0.273-2.026	0.563
NLR						
≤3.26 >3.26	Reference 1.637	0.841-3.189	0.147	Reference 2.265	1.138-4.508	0.020
Thyroid irAEs						
No	Reference		0.031	Reference		0.024
Yes	0.447	0.215-0.931		0.424	0.201-0.893	

NLR, neutrophil: lymphocyte ratio.

### 3.3 The association analysis for clinical characteristics and thyroid irAEs

The thyroid irAEs identified in this study were mild (grade 1–2). The association between thyroid irAEs and clinical characteristics at baseline was analyzed subsequently by univariate analysis. Higher BMI (*p* = 0.005), increased eosinophil count (*p* = 0.014), increased LDH (*p* = 0.008), higher TSH (*p* = 0.001), HCC (*p* = 0.001) and increased ADA (*p* = 0.001) were linked with thyroid irAEs occurrence. The multivariate analysis, shown in **Table 4**, indicated that higher BMI [odds rate (OR) = 5.095, 95%CI: 1.316 to 19.730, *p* = 0.018], increased eosinophil count (OR = 7.553, 95%CI: 1.940 to 29.400, *p* = 0.004), increased ADA (OR = 4.756, 95%CI: 1.147 to 19.729, *p* = 0.032), higher TSH (OR = 3.540, 95%CI: 1.007 to 12.440, *p* = 0.049), lower ECOG PS level (OR = 0.221, 95%CI: 0.054 to 0.899, *p* = 0.035), and positive TPOAb (OR = 11.326, 95%CI: 1.637 to 78.355, *p* = 0.014) were independently associated with the occurrence of thyroid irAEs.

**Table 4:** Univariate and multivariate analysis of thyroid immune-related adverse events (irAEs) with Logistic regression models.

	Univariate analysis			Multivariate analysis		
	OR	95% CI	*p*	OR	95% CI	*p*
Sex						
female	Reference		0.676	Reference		0.455
male	0.858	0.418-1.7760		1.698	0.423-6.809	
Age (years)						
≤60	Reference		0.202	Reference		0.833
>60	0.622	0.300-1.291		0.878	0.263-2.936	
BMI (kg/m^2^)						
≤21.06	Reference		0.005	Reference		0.018
>21.06	3.062	1.396-6.719		5.095	1.316-19.730	
ECOG PS						
≤1	Reference		0.433	Reference		0.035
>1	0.750	0.365-1.540		0.221	0.054-0.899	
Tumor Types						
Gastric Cance	Reference			Reference		
HCC	5.950	2.387-14.833	0.001	5.467	0.884-33.824	0.068
Esophageal squamous cell carcinoma	0.968	0.769-5.037	0.158	0.816	0.128-5.218	0.830
TPOAb						
Negative	Reference		0.071	Reference		0.014
Positive	2.454	0.927-6.495		11.326	1.637-78.355	
TGAb						
Negative	Reference		0.416	Reference		0.451
Positive	1.500	0.565-3.981		0.458	0.060-3.484	
NLR						
≤3.26	Reference		0.100	Reference		0.711
>3.26	1.853	0.888-3.868		1.288	0.338-4.906	
PLR						
≤286.48	Reference		0.383	Reference		0.076
>286.48	1.484	0.611-3.603		3.999	0.863-18.528	
Eosinophil count)						
≤0.20 × 10^9^/L	Reference		0.014	Reference		0.004
>0.20 × 10^9^/L	3.026	1.251-7.315		7.553	1.940-29.400	
ADA (U/L)						
≤14.05	Reference		0.001	Reference		0.032
>14.05	4.304	1.952-9.492		4.756	1.147-19.729	
LDH (U/L)						
≤173.50	Reference		0.008	Reference		0.513
>173.50	3.042	1.336-6.927		1.552	0.416-5.792	
Baseline TSH (mIU/L)						
≤2.205	Reference		0.001	Reference		0.049
>2.205	4.040	1.860-8.776		3.540	1.007-12.440	

## 4 Discussion

Thyroid irAEs were the most prevalent PD-1 inhibitors related irAEs including hypothyroidism, hyperthyroidism, biphasic thyroiditis, and central hypothyroidism, with hypothyroidism comprises the majority (9). Our study revealed thyroid irAEs in 47 out of 129 patients (36.4%), which is comparable to previous reports (42%) (4). Despite the occurrence of thyroid irAEs, most patients exhibited a low fatality rate and manifested symptoms, such as arrhythmia, myxedema, and marasmus (10). Timely monitoring of thyroid function is crucial to prevent thyroid dysfunction in cancer patients receiving the ICI treatment and maintain sustained therapeutic effects. Patients with thyroid irAEs tended to a longer OS and a trend of longer PFS than those without thyroid dysfunction, which was also comparable to the previous reports (6).

Patients with HCC were more prone to thyroid thyroid irAEs compared with other cancer types, although this conclusion needs to be validated in a larger population. The liver is the site for thyroid hormone synthesis and metabolism, HCC can initiate more synthesis and secretion of thyroxine binding globulin, which contributes to the occurrence of hypothyroidism by augmented thyroxine binding globulin-T4 binding (11, 12). In addition, signaling pathways, such as TGF-β, Wnt, and Hedgehog, midiate inflammation and fibrosis of HCC, which can regulate the metabolism of thyroid hormones. Liver abnormalities are more likely to induce the abnormalities of thyroid hormone metabolism, which may be one of the reasons why patients with HCC are more prone to thyroid irAEs (13).

Our investigation identified a higher baseline level of ADA (>14.05 U/L) as an independent biomarker associated with thyroid irAEs. ADA, a key enzyme involved in purine metabolism and immune system regulation, has been associated with thyroid autoimmune diseases at the transcriptional level (14). It can activate T lymphocytes (15) as well as alleviate immunosuppressive effects by degrading adenosine (16). The adenosine receptors on the dendritic cells (DCs) can interact with CD26 on the surface of T cells to mediate the CD4+ T cell differentiation (17), DCs deliver antigens to CD4+ T cells so as to stimulate the production of thyroid related antibodies, thereby destroying the thyroid follicular cells (18). In addition, the number of DCs increased significantly when thyroid autoimmune response occurred to aggravate the immune response (19). ADA might modify the thyroid irAEs by stimulating DCs and T cells through the adenosine receptors on DCs. The increasing of ADA after ICI treatment especially in thyroid irAEs patients reminds us to pay attention to the change of thyroid function.

We identified BMI (>21.06 kg/m^2^), ECOG PS (≤1) and eosinophil count (>0.20 × 10^9^/L) as associated factors for thyroid irAEs. Consistent report demonstrated that a higher BMI is associated with increased incidence of irAEs and improved outcomes in various cancers such as NSCLC, melanoma, renal cell carcinoma, head and neck tumors, and epithelial urothelial carcinoma (20, 21). Additionally, a study involving approximately 19% of patients with gastrointestinal tumors suggested a correlation between higher BMI and increased risk of overt thyroid dysfunction in ICI-treated cancers (21). Our study corroborated these findings, demonstrating that patients with gastrointestinal tumors and higher BMI are more susceptible to thyroid irAEs. One plausible explanation for this association is that obesity induces systemic inflammation and compromises immune response through modulation of leptin secretion, which is known to correlate with PD-1 expression in CD8+ T cells (22).

In our previous study, we observed that a lower ECOG PS was associated with a better prognosis (23). However, the relationship between ECOG PS and irAEs remains contentious due to the limited data. While ECOG PS (≤1) was identified as an independent risk factor for irAEs in patients with NSCLC who received ICI treatment (24), however, another study found no significant association between ECOG PS and irAEs for patients with metastatic renal cell carcinoma who received ICI treatment (25). This potential association between ECOG PS and thyroid irAEs should be re-evaluated in a larger population.

Additionally, higher eosinophil count (>0.20 × 10^9^/L), higher level of TSH (>2.205mIU/L) and positivie TPOAb were identified as baseline factors for thyroid irAEs association in gastrointestinal tumors, which was also consistent with previous findings in solid tumors (26–28).

## 5 Conclusions

Increased baseline level of ADA was associated with thyroid irAEs occurrence in patients with advanced gastrointestinal tumors who received ICI treatment. In the case of abnormal ADA, attention should be paid to the risk of thyroid irAEs.

## Data availability statement

The original contributions presented in the study are included in the article/supplementary materials, further inquiries can be directed to the corresponding author/s.

## Ethics statement

The studies involving humans were approved by The Ethics Committee of The Fourth Hospital of Hebei Medical University. The studies were conducted in accordance with the local legislation and institutional requirements. The ethics committee/institutional review board waived the requirement of written informed consent for participation from the participants or the participants’ legal guardians/next of kin because This study was retrospective and analyzed by existing data, waiver of informed consent was applied for the patient.

## Author contributions

NX: Conceptualization, Funding acquisition, Methodology, Validation, Writing – original draft, Writing – review & editing. JL: Data curation, Formal analysis, Investigation, Software, Writing – review & editing. LH: Formal analysis, Project administration, Validation, Visualization, Writing – review & editing. YZ: Resources, Supervision, Visualization, Writing – review & editing. HM: Data curation, Resources, Writing – review & editing. FW: Conceptualization, Supervision, Writing – review & editing. ZG: Conceptualization, Funding acquisition, Resources, Supervision, Validation, Writing – review & editing.
